# Optimizing MATRix as remission induction in PCNSL: de-escalated induction treatment in newly diagnosed primary CNS lymphoma

**DOI:** 10.1186/s12885-022-09723-w

**Published:** 2022-09-10

**Authors:** Julia Wendler, Christopher P. Fox, Elke Valk, Cora Steinheber, Heidi Fricker, Lisa K. Isbell, Simone Neumaier, Jessica Okosun, Florian Scherer, Gabriele Ihorst, Kate Cwynarski, Elisabeth Schorb, Gerald Illerhaus

**Affiliations:** 1 Clinic of Hematology, Oncology and Palliative Care, Klinikum Stuttgart, Kriegsbergstraße 60, 70174 Stuttgart, Germany; 2 Russell Centre for Clinical Haematology, Nottingham University Hospitals NHS Trust, City Campus, Hucknall Road, Nottingham, NG5 1PB UK; 3 Stuttgart Cancer Center - Tumorzentrum Eva Mayer-Stihl, Klinikum Stuttgart, Kriegsbergstraße 60, 70174 Stuttgart, Germany; 4 Department Medicine I, Medical Center-University of Freiburg, Faculty of Medicine, University of Freiburg, Hugstetter Straße 55, 79106 Freiburg, Germany; 5 Centre for Haemato-Oncology, Barts Cancer Institute, Queen Mary University of London, London, UK; 6 Clinical Trials Unit, Faculty of Medicine and Medical Center, University of Freiburg, Elsässer Straße 2, 79110 Freiburg, Germany; 7 Department of Haematology, University College of London Hospitals, NHS Foundation Trust, London, UK

**Keywords:** Primary central nervous system lymphoma (PCNSL), High-dose chemotherapy (HCT), Autologous stem cell transplantation (ASCT), Toxicity, De-escalation

## Abstract

**Background:**

Primary diffuse large B-cell lymphoma (DLBCL) of the central nervous system (PCNSL) is a rare disorder with an increasing incidence over the past decades. High-level evidence has been reported for the MATRix regimen (high-dose methotrexate (HD-MTX), high-dose AraC (HD-AraC), thiotepa and rituximab) followed by high-dose chemotherapy and autologous stem cell transplantation (HCT-ASCT) supporting this approach to be considered a standard therapy in newly diagnosed PCNSL patients ≤ 70 years. However, early treatment-related toxicities (predominantly infectious complications), occurring in up to 28% per MATRix cycle, diminish its therapeutic success. Furthermore, sensitivity to first-line treatment is an independent prognostic factor for improved overall survival (OS) in PCNSL. Thus, patients achieving early partial remission (PR) after 2 cycles of MATRix might be over-treated with 4 cycles, in the context of consolidation HCT-ASCT.

**Methods:**

This is an open-label, multicentre, randomized phase III trial with two parallel arms. 326 immunocompetent patients with newly diagnosed PCNSL will be recruited from 37 German, 1 Austrian and 12 UK sites. Additional IELSG (International Extranodal Lymphoma Study Group) sites are planned. The objective is to demonstrate superiority of a de-escalated and optimised remission induction treatment strategy, followed by HCT-ASCT. Randomization (1:1) will be performed after completion of all screening procedures. Patients in Arm A (control treatment) will receive 4 cycles of MATRix. Patients in Arm B (experimental treatment) will receive a pre-phase (R/HD-MTX), followed by 2 cycles of MATRix. Patients in both arms achieving PR or better will proceed to HCT-ASCT (BCNU, thiotepa). The primary endpoint of the study is event-free-survival (EFS), defined as time from randomization to premature end of treatment due to any reason, lymphoma progression or death whichever occurs first. Secondary endpoints include OS, progression free survival (PFS), toxicity, neurocognitive impairment and quality of life. Minimal follow-up is 24 months.

**Discussion:**

Current treatment options for PCNSL in patients ≤ 70 years have improved remarkably over recent years. However, the potential efficacy benefits are offset by an increased incidence of short-term toxicities which can impact on treatment delivery and hence on survival outcomes. In patients ≤ 70 years with newly diagnosed PCNSL addressing the need to reduce treatment-related toxicity by de-escalating and optimising the induction phase of treatment, is a potentially attractive treatment strategy.

**Trial registration:**

German clinical trials registry DRKS00022768 registered June 10^th^, 2021.

**Supplementary Information:**

The online version contains supplementary material available at 10.1186/s12885-022-09723-w.

## Background

Primary diffuse large B-cell lymphoma (DLBCL) of the central nervous system (PCNSL) constitutes a rare disorder restricted to the cerebral parenchyma, leptomeninges, eyes or spinal cord observed with increasing incidence over recent decades, particularly in immunocompetent individuals [1]. With a median survival of three months in untreated individuals, prognosis of PCNSL is poor [2].

The randomized IELSG32 trial of the International Extranodal Lymphoma Study Group (IELSG) provides high-level evidence for the MATRix regimen comprising high-dose methotrexate (HD-MTX), high-dose cytarabine (HD-AraC), thiotepa and the CD20-antibody rituximab in patients aged 70 years or younger, which was demonstrably superior to the combination of HD-MTX/HD-AraC or rituximab/HD-MTX/HD-AraC [3]. Latest follow-up data show 7-year PFS of 52% and 7-year OS of 56% after 4 cycles of MATRix in comparison to PFS rates of 20% and 29% and OS rates of 26% and 37% when only HD-MTX/AraC or rituximab/HD-MTX/AraC were received (Illerhaus, Ferreri et al., abstract from EHA and ICML 2021 respectively). Due to its excellent 2-year OS and PFS of 69% and 61%, the MATRix regimen, followed by HCT-ASCT, is considered a standard induction strategy in many countries [3, 4]. Recently, updated follow-up data show ongoing treatment success following MATRix and HCT-ASCT with 7-year OS and PFS rates of 70% (Illerhaus, Ferreri et al., abstract from EHA and ICML 2021 respectively). In a number of clinical trials and retrospective studies HCT-ASCT comprising carmustine (BCNU) and thiotepa has been shown to be feasible and highly effective as consolidation therapy [5–9].

However, early treatment-related toxicities, commonly occurring in the 1^st^ cycle and frequently infectious in origin, limit the therapeutic success of MATRix. In an international retrospective analysis investigating the MATRix regimen in routine clinical practice, infectious toxicities were reported in 11–28% of each MATRix cycle. Within the 1^st^ cycle of MATRix the most severe toxicities occurred, with 6% of patients requiring admission to the intensive care unit in comparison to only one admission during cycle 2–4 of MATRix [10]. It is likely that the incidence of infectious toxicities within the 1^st^ cycle of MATRix is associated with impairment of performance status and neurocognitive disability (due to PCNSL), together with the immunosuppressive effects of corticosteroid exposure, frequently prescribed during the PCNSL diagnostic pathway.

A few randomised trials provide evidence for the feasibility of a pre-phase treatment prior to the 1^st^ chemotherapy cycle in elderly patients suffering from systemic DLBCL. A pre-phase treatment was implemented in the respective protocols in order to improve the performance status and prevent the so called ‘first-cycle effect’ – described as deepest nadir of absolute neutrophil count, longest duration of neutropenia as well as highest rate of therapy-associated deaths and subsequently ameliorate treatment delivery [11, 12].

Furthermore, sensitivity to first-line therapy, which is defined as achieving at least PR during or at the end of therapy, has been shown to be an independent prognostic factor for improved OS in PCNSL [13]. Thus, patients achieving therapeutic response early in the course of therapy, might be over-treated by 4 cycles of MATRix induction treatment.

In the subgroup of elderly PCNSL patients, attenuated remission induction has already been successfully applied in the MARiTA pilot trial demonstrating a high number of patients reaching consolidation treatment by reducing short-term toxicities and is associated with promising results regarding OS and PFS [14]. The attenuation of remission induction comprising HD-MTX, HD-AraC and rituximab, followed by thiotepa-based HCT-ASCT, is based on the fact that this elderly subgroup is not expected to tolerate intensive immunochemotherapy and is evaluated in a larger cohort in the ongoing MARTA trial (DRKS00011932).

Despite advances in treatment outcome in PCNSL patients aged 70 years and younger with 4 cycles of the MATRix regimen followed by HCT-ASCT, there is a great need to reduce treatment related toxicities without losing efficacy. We therefore propose this multicentric phase III trial investigating efficacy, feasibility and safety of a de-escalated induction therapy followed by HCT-ASCT in newly diagnosed PCNSL patients.

## Methods

### Study design

This is an open-label, multicentric, randomized phase III trial with two parallel arms. The study design was authorized by the leading ethics committee (Ethik-Kommission der Landesärztekammer Baden-Württemberg in Stuttgart, Germany) as well as the local ethics committees of the participating centres. The protocol was also subject to approval by the competent authorities as mandatory by federal law. All participants have to provide written informed consent. The trial was assigned the EudraCT number 2018–002,115-96 and is registered at German clinical trials registry (DRKS00022768, registration date June 10^th^, 2021).

### Study objectives and endpoints

The primary objective of this trial is to demonstrate superiority (in terms of EFS) of an optimised induction treatment strategy, comprising a pre-phase (R/HD-MTX) and abbreviated delivery of the MATRix regimen (2 cycles), compared to standard delivery of the MATRix regimen (4 cycles), followed by HCT-ASCT in both arms. The primary endpoint EFS is defined as time from randomization to premature end of treatment due to any reason, lymphoma progression or death, whichever occurs first. Patients will be censored at the time of last follow-up if no event of interest has occurred.

The secondary endpoints include OS, PFS, remission rate (CR and PR) prior to consolidation treatment (measured at response assessment II) and after consolidation treatment (measured 30 days after ASCT) and the rate of patients proceeding to consolidation therapy. Tumour response will be assessed by gadolinium-enhanced brain MRI according to the International PCNSL Collaborative Group’s (IPCG) response criteria [15] and will be evaluated by central independent radiological review, not engaged in the conception of the study and blinded to the treatment assignment. Furthermore, tumour size, location(s) and manifestation (singular/multiple) will be assessed. In case of multiple tumours, one reference tumour will be analysed.

Secondary safety endpoints include (serious) adverse events, toxicity (according to National Cancer Institute’s Common Terminology Criteria for Adverse Events (NCI-CTCAE) v5.0), quality of life (QoL) (measured by EORTC QLQ-C30 and -BN20) between both treatment arms as well as rate of unplanned hospital admissions and length of hospital stays. Additionally, neurocognitive impairment will be measured by Montreal Cognitive Assessment (MoCA) and Trail-Making-Test-A and -B (TMT-A/B) as well as a neuro-psychological test battery comprising WAIS (Wechsler Adult Intelligence Scale) III—counting test, Hopkins Verbal Learning Test, Brief Test of Attention, Grooved Pegboard Test and a verbal fluency test. In order to expand the neurocognitive domains covered, but keep the burden as low as possible on the patients, 50% of patients each will be assigned to ‘version 1’ or ‘version 2’ of the neuropsychological test battery in a separate randomization process (1:1). ‘Version 1’ additionally comprises the Rey-Osterrieth-Complex-Figure-Test and ‘version 2’ the WAIS III—subtest similarities. The objective of this procedure is to assess the feasibility of the altered neuropsychological test battery.

### Eligibility criteria

Eligible are immunocompetent patients with newly-diagnosed PCNSL, aged 18–65 years irrespective of Eastern Cooperative Oncology Group Performance Status (ECOG PS) or 66–70 years with an ECOG PS ≤ 2. Further details on inclusion and exclusion criteria are summarized in Table 1.

**Table 1:** Inclusion and exclusion criteria

Inclusion criteria	1. Immunocompetent patients with newly diagnosed primary diffuse large B-cell lymphoma (DLBCL) of the central nervous system (CNS) 2. Male or female patients aged 18–65 years irrespective of ECOG PS or 66–70 years with ECOG PS ≤ 2 3. Histologically or cytologically assessed diagnosis of B-cell lymphoma by local pathologist. Diagnostic sample obtained by stereotactic or surgical biopsy, CSF cytology examination or vitrectomy 4. Disease exclusively located in the CNS 5. At least one measurable lesion 6. Previously untreated patients (previous or ongoing steroid treatment admitted) 7. Negative pregnancy test 8. Written informed consent obtained according to international guidelines and local laws by patient or authorized legal representative in case patient is temporarily legally not competent due to his or her disease 9. Ability to understand the nature of the trial and the trial related procedures and to comply with them
Exclusion criteria	1. Congenital or acquired immunodeficiency including human immunodeficiency virus (HIV) infection and previous organ transplantation 2. Systemic lymphoma manifestation (outside the CNS) 3. Primary vitreoretinal lymphoma without manifestation in the brain parenchyma or spinal cord 4. Previous or concurrent malignancies with the exception of surgically cured carcinoma in situ of the cervix, carcinoma of the skin or other kinds of cancer without evidence of disease for at least 5 years 5. Previous Non-Hodgkin lymphoma at any time 6. Inadequate renal function (creatinine clearance < 60 ml/min) 7. Inadequate bone marrow, cardiac, pulmonary or hepatic function according to investigator´s decision 8. Active hepatitis B or C disease 9. Concurrent treatment with other experimental drugs or participation in an interventional clinical trial with study medication being administered within the last 30 days before the start of this study 10. Third space fluid accumulation > 500 ml 11. Hypersensitivity to study treatment or any component of the formulation 12. Taking any medications that are likely to cause interactions with the study medication 13. Known or persistent abuse of medication, drugs or alcohol 14. Active COVID-19-infection or non-compliance with the prevailing hygiene measures regarding the COVID-19 pandemic 15. Patients without legal capacity who are unable to understand the nature, significance and consequences of the trial and without designated legal representative 16. Previous participation in this trial 17. Persons who are in a relationship of dependency/ employment with the sponsor and/ or the investigator 18. Any familial, sociological or geographical condition potentially hampering compliance with the study protocol and follow-up schedule 19. Current or planned pregnancy, nursing period 20. For fertile patients: Failure to use one of the following safe methods of contraception: intra-uterine device or hormonal contraception in combination with a mechanical method of contraception

### Randomization methodology

Randomization will be carried out stratified by site, in blocks of variable length in a ratio of 1:1. The trial biometrician will provide the randomization lists which will be uploaded into secuTrial® by the data management. The randomization sequence will be generated by validated programs based on the Statistical Analysis System (SAS®). Documentation of block length will be done separately and not disclosed to the sites [16].

A randomization form will be created in secuTrial®. Performance of randomization will be electronically at the study site in secuTrial® to guarantee concealment of the next treatment allocation. Randomization will take place once all screening procedures are completed and all inclusion/exclusion criteria are verified, immediately before starting either standard induction treatment or de-escalated induction treatment. Stratification by study site will be performed. Due to a large number of centres with presumably few patients, other relevant factors (age < 60 years vs. ≥ 60 years, ECOG PS 0/1 vs. ≥ 2) will not be considered as stratification factors in the randomization process, but will be integrated as covariates in the primary analysis.

A second, separate randomization list will be prepared independently in order to assign patients to ‘version 1’ or ‘version 2’ of the neuropsychological test battery. Randomization will be carried out in blocks of variable length in a ratio of 1:1, stratified by study site. This procedure is applied due to administrative reasons and does not aim for a comparison between patient groups.

### Treatment schedule

**Fig. 1:**
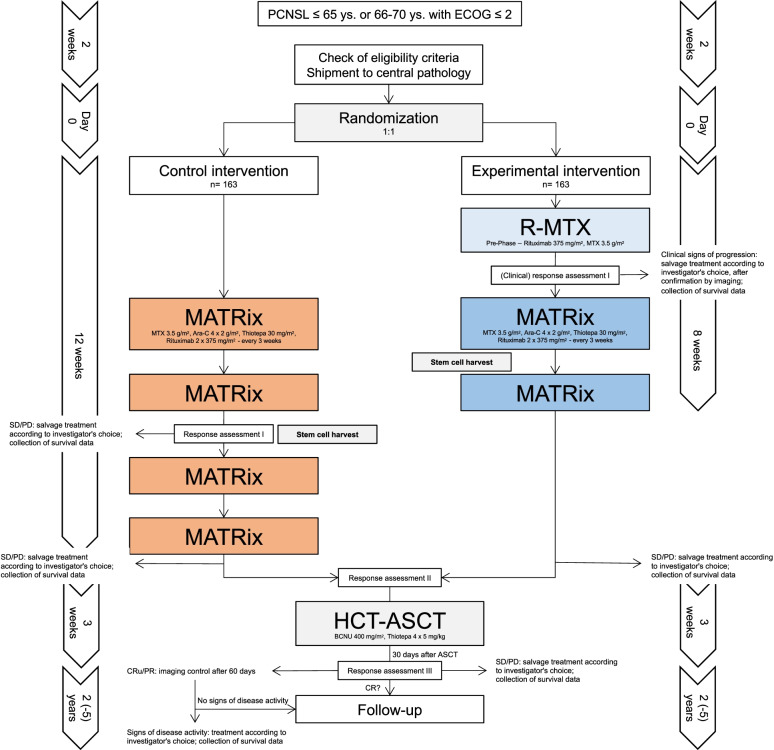
Intervention Scheme

The treatment schedule is shown in Fig. 1 (Intervention scheme). Patients will either receive the standard induction treatment (Arm A) comprising 4 cycles of the MATRix regimen or the experimental induction treatment (Arm B) comprising a pre-phase treatment with R/HD-MTX followed by 2 cycles of the MATRix regimen.

### Induction treatment

#### Arm A (control intervention)

Induction treatment in Arm A comprises 4 cycles (every 3 weeks) of the MATRix regimen [16]. Rituximab will be administered intravenously at 375 mg/m^2^ on day 0 and 5 of each cycle. HD-MTX will be administered intravenously at 0.5 g/m^2^ in 15 min and then 3 g/m^2^ over a period of 3 h on day 1 of each cycle. HD-AraC will be administered intravenously at 2 g/m^2^ over a period of 1 h, twice a day (every 12 h) on 2 consecutive days (day 2 and 3) of each cycle and thiotepa will be administered intravenously at 30 mg/m^2^ over 30 min on day 4 of each cycle. Stem cell harvest will be performed after cycle 2 of MATRix according to local standard procedure.

#### Arm B (experimental intervention)

Induction treatment in Arm B comprises a pre-phase therapy with rituximab and HD-MTX, followed by 2 cycles of the MATRix regimen (every 3 weeks). Rituximab will be administered intravenously at 375 mg/m^2^ on day 0 and HD-MTX will be given at 0.5 g/m^2^ in 15 min and then 3 g/m^2^ over a period of 3 h on day 1 during the pre-phase therapy. In the absence of clinical signs of progression, treatment will proceed with 2 cycles of the MATRix regimen (every 3 weeks) on day 10–14 of the pre-phase therapy. For specific details on administration please refer to the section above. Stem cell harvest will be performed after cycle 1 of MATRix according to local standard procedure.

### Consolidation treatment

All patients will receive HCT comprising administration of carmustine (BCNU) at 400 mg/m^2^ intravenously over 1 h on day -6 and of thiotepa intravenously at 5 mg/kgBW over a period of 2 h, twice a day (every 12 h) on 2 consecutive days (day -5 and day -4). Autologous stem cell reinfusion will be performed on day 0, according to standard procedures. Busulfan, given intravenously at 3.2 mg/kg over 3 h once a day on 2 consecutive days (day -8 to -7) before ASCT, can be used if carmustine (BCNU) is not available at the study site.

### Assessments and follow-up

The following parameters will be gathered at each visit: ECOG PS, vital signs, thorough physical and neurological examination, laboratory tests and adverse events.

Gadolinium-enhanced brain MRI will be performed after cycle 2 and 4, as well as on day 25–35 after HCT-ASCT in Arm A and after cycle 4 and on day 25–35 after HCT-ASCT in Arm B. Regarding Arm B, response assessment by imaging will only be performed in case of clinical signs of progression after pre-phase therapy. During the subsequent 2-year follow-up period gadolinium-enhanced brain MRI will be performed every 3 months. Afterwards, further control examinations are recommended to be performed every 6 months during years 3 to 5 and once yearly thereafter. Tumour size and location(s) will be assessed only at screening and in case of progressive disease (PD)). Tumour number (singular/multiple) will only be assessed at screening.

Moreover, a comprehensive translational research program will be implemented within this trial in order to improve the understanding of the molecular factors driving lymphomagenesis and disease aggressiveness and to identify and validate biomarkers that predict patient outcome and, in the future, allow improved risk stratification. First, we plan to undertake a comprehensive analyses of the pre-treatment tumour biopsies to define molecular subgroups of patients and identify biologically relevant (epi-)genetic aberrations. In addition, we will perform a broad evaluation of circulating tumour DNA (ctDNA) in blood plasma and cerebrospinal fluid at various disease milestones, as ctDNA has shown great potential as a prognostic biomarker in a retrospective series (Mutter et al., Oral abstract ICML 2021).

Further information on assessments during the trial and the follow-up period are shown in the appendix (see Additional File 1a and b: trial flow chart).

### Sample size calculation

The sample size calculation of this trial is based on the primary endpoint EFS. In the international IELSG32 trial, the 2-year PFS rate in the best arm was 61% [4]. An EFS rate similar to PFS in the IELSG32 trial is expected in the standard intervention group. It is noted that EFS includes more events than PFS. We aim to increase the 2-year EFS by at least 12% to 73% of patients being event free after 2 years receiving the experimental intervention. This corresponds to a hazard ratio of 0.636. Then, 153 events need to be observed to detect this difference with 80% power at a 2-sided significance level of 5%. Assuming an exponential model for survival, an accrual period of 4 years and an additional follow-up time of 2 years, 292 patients (146 per arm) need to be included in the analysis (calculated with nQuery 7.0). Assuming a dropout rate of around 10%, we aim to include 326 patients in this trial.

### Statistical analysis

The primary analysis will be performed according to the intention-to-treat principle and based on the full analysis set (FAS). All randomized patients in whom therapy after randomization was initiated will be included in the FAS. Patients will be analysed according to their randomisation, regardless of refusal or discontinuation of therapy or other protocol deviations known [16].

The primary endpoint EFS will be analysed with a Cox proportional hazards regression model. This regression model will include randomized treatment and the following independent variables for adjustment: the stratification variable trial centre, further: age (< 60 years vs. ≥ 60 years) and ECOG PS (0/1 vs. ≥ 2).

The treatment effect will be described by the estimated hazard ratio (experimental arm vs. standard arm) from this model and will be presented with a two-sided 95% confidence interval. The test of the difference between the experimental arm and the control arm at a two-sided significance level of 5% will be based on the corresponding asymptotic two-sided 95% confidence interval from the Cox regression model.

Additionally, the EFS rates will be estimated by the Kaplan–Meier method.

The secondary endpoints PFS and OS will be analysed in the same way as described for EFS. The endpoints remission rate of patients reaching consolidation and rate of remission prior (measured at response assessment II) and after consolidation treatment (measured 30 days after ASCT) will be analysed as the dependent variable of a logistic regression model with treatment assignment and the same covariates as in the primary analysis of EFS as independent variables. The crude proportion of patients achieving CR after the end of treatment in each treatment group will be calculated in the FAS, and will be presented with accompanying two-sided 95% confidence intervals. Missing values will be counted as non-responders.

Sensitivity analyses will be performed by additionally including other relevant prognostic variables: gender, LDH level (≤ vs. > above upper normal limit), presence of deep brain lesions (yes vs. no) and protein count of cerebrospinal fluid (≤ vs. > above upper normal limit), to adjust for potential confounding in the regression models proposed above for the primary and the secondary endpoints.

The number of unplanned hospital admissions and the length of hospital stays (number of days in hospital) will be analysed descriptively by treatment arm.

QoL and neurotoxicity measures will be analysed descriptively by treatment arm and time point. Changes from baseline (screening assessment) will be described.

Safety analyses will be conducted in the safety population. Patients in the safety population are analysed as belonging to the treatment arm defined by treatment received. Patients are included in the respective treatment arm, if treatment was initiated.

### Quality assurance and safety

The data management will be performed with secuTrial® (https://www.secutrial.com/). Data will be checked during data entry by so-called edit checks. The data will be reviewed for completeness, plausibility, consistency, and regarding protocol violations and other distinctive problems (e.g. cumulative missing data) using study-specific reports programmed and generated with secuTrial® or SAS software. All programs which can be used to influence the data or data quality will be validated.

### Data monitoring committee

The course of the trial will be reviewed by an independent Data Monitoring Committee (DMC). Members of the DMC are two medical scientists as well as one statistician with longstanding experience in clinical trials. The DMC will make recommendations for study continuation, modification or discontinuation to the coordinating or principal investigator. The DMC members will receive the development safety update reports (DSURs) and will be informed about patient recruitment, adherence to the protocol, observed serious adverse events and deaths in regular meetings.

## Discussion

Current treatment options for PCNSL in patients ≤ 70 years have remarkably improved over recent years. Within the IELSG32 trial, we have demonstrated the MATRix regimen comprising rituximab, HD-MTX, HD-AraC and thiotepa as induction treatment to be safe and highly effective [3]. Regarding consolidation treatment, HCT-ASCT has been investigated in several phase II and III trials [5, 7, 16], demonstrating encouraging response and OS rates. In comparison to whole brain radiation therapy, HCT-ASCT shows beneficial results concerning long-term neurotoxicity [4].

On this account, 4 courses (administered every 3 weeks) of the MATRix regimen, followed by HCT-ASCT is widely accepted as standard therapy in newly diagnosed PCNSL aged ≤ 70 years and therefore is implemented in the control intervention.

Yet, these outcome benefits are offset by an increased incidence of acute and severe toxicity, especially during the 1^st^ course of induction treatment with the MATRix regimen, which comprise mainly infectious complications [10]. Addressing the need to reduce serious treatment-related toxicities, we have devised a de-escalated induction treatment comprising a pre-phase treatment with rituximab and HD-MTX followed by an abbreviated delivery of the MATRix regimen (only 2 cycles) in the experimental intervention. This optimised approach is consistent with experimental approaches for elderly patients suffering from systemic DLBCL, in which a pre-phase treatment has been shown to be feasible [11, 12]. Regarding the elderly subgroup of PCNSL patients, a reduction of treatment intensity in induction treatment has been successfully applied providing reduced toxicity, resulting in a majority of patients proceeding to a potentially curative consolidation treatment (HCT-ASCT) in a pilot trial [14].

Moreover, sensitivity to first-line therapy, which is defined as achieving at least PR during or at the end of therapy, was an independent prognostic factor for improved OS in PCNSL [13], suggesting patients achieving PR or CR in interim staging by gadolinium-enhanced brain MRI may be overtreated by the standard induction treatment comprising 4 cycles of the MATRix regimen. This supports the rationale for de-escalating the induction phase of treatment, with 2 further courses of the MATRix regimen after an attenuated first treatment cycle (pre-phase).

Within this trial, we aim to demonstrate the superiority of an optimised induction treatment comprising an abbreviated delivery of only 2 cycles of the MATRix regimen in addition to a pre-phase (R/HD-MTX) compared to the established standard therapy with 4 cycles of MATRix, each followed by consolidating HCT-ASCT, in terms of EFS. The implementation of independent pathologic and radiologic review as described herein meets high-level methodical and quality standards.

The multicentre OptiMATe trial is expected to generate results which provide high-level evidence regarding safety and effectiveness of an optimised induction treatment regimen followed by HCT-ASCT in newly diagnosed PCNSL patients aged ≤ 70 years.

## Supplementary Information

**Additional file 1: a**. Flow Chart - control intervention (Arm A). **b**. Flow Chart - experimental intervention (Arm B)

## Data Availability

As this is a study protocol report data sharing is not applicable to this article as no datasets were analysed during the current study.

## Abbreviations

ASCT: Autologous stem cell transplantation
BCNU: Carmustine
BW: Bodyweight
CNS: Central Nervous System
CR: Complete remission
ctDNA: Circulating Tumour Deoxyribonucleic Acid
CTU: Clinical Trials Unit
D: Day
DMC: Data Monitoring Committee
DLBCL: Diffuse large B-cell lymphoma
ECOG: Eastern Cooperative Oncology Group
EFS: Event Free Survival
EHA: European Hematology Association
EORTC QLQ-C30: European Organization for Research and Treatment of Cancer Questionnaire Core-30
EORTC QLQ-BN20: European Organization for Research and Treatment of Cancer Questionnaire Brain Tumour module 20
FAS: Full Analysis Set
HD-AraC: High-dose cytarabine
HD-MTX: High-dose methotrexate
H(D)CT: High-dose chemotherapy
HIV: Human immunodeficiency virus
ICML: International Conference on Malignant Lymphoma
IELSG: International Extranodal Lymphoma Study Group
IPCG: International PCNSL Collaborative Group
MATRix: HD-MTX, HD-AraC, Thiotepa, Rituximab
MoCA: Montreal Cognitive Assessment
MRI: Magnetic Resonance Imaging
NCI-CTCAE: National Cancer Institute—Common toxicity criteria for adverse events
OS: Overall survival
PCNSL: Primary central nervous system lymphoma
PET: Positron Emission Tomography
PD: Progressive disease
PFS: Progression free survival
PR: Partial remission
PS: Performance status
QoL: Quality of life
SAS: Statistical Analysis System
SD: Stable disease
TMT-A/-B: Trail Making Test A/B
WAIS: Wechsler Adult Intelligence Scale
