# Supplementary material for: Optimizing MATRix as remission induction in PCNSL: de-escalated induction treatment in newly diagnosed primary CNS lymphoma
Source: BMC Cancer. 2022 Sep 10;22:971. doi: 10.1186/s12885-022-09723-w (PMC9464101; doi:10.1186/s12885-022-09723-w)
Supplement: Supplementary file 1 — Additional file 1: a. Flow Chart - control intervention (Arm A). b. Flow Chart - experimental intervention (Arm B) [file 12885_2022_9723_MOESM1_ESM.zip › FlowChart_ArmAR2.pdf]

Table 1 Visit schedule and assessments – Flowchart: Control Treatment – Arm A

PERIODS	Name	SCREENING		TREATMENT – ARM A							EOT	FOLLOW-UP	
	Duration	14 days		16 weeks								2(-5) years	
VISITS	Title in eCRF	Screening		Visit 1	Visit 2	RA I	Visit 3	Visit 4	RA II	Visit 5	RA III	FU RA III – Yr 2	FU Yr 3-5[2]
	Time / Section	d-14 until d0	d0	d0-5 of cycle 1[1]**	d0-5 of cycle 2[1]**	d18-20 of cycle 2[1]	d0-5 of cycle 3[1]**	d0-5 of cycle 4[1]**	d18-20 of cycle 4[1]	d-6-0 of HCT[1,xx]	d25-35 after ASCT	*Every 3 mo*	*Every 6 mo*
**Informed Consent[3]**	15.3	X											
**Inclusion/ Exclusion Criteria**	4.2,4.3	X											
**Demographics, Medical History**	7.5.1,7.5.2	X											
**Pregnancy Test[4]**	7.5.3	X								X	X		
**Randomization**	5.2.		X										
**Physical and neurological examination***	7.5.4	X		X	X		X	X		X	X	X	X
**Vital signs***/ body height and weight**	7.5.5	X		X	X		X	X		X	X	X	X
**Performance status (Karnofsky and ECOG)**	7.5.6	X		X	X		X	X		X	X	X	X
**Laboratory tests (haematology[5], clinical chemistry[6])*****	7.5.9	X		X	X		X	X		X	X	X	X
**Creatinine, estimated GFR (MDRD) [6]**	7.5.9	X		X	X		X	X		X			
**LDH[6]**	7.5.9	X											
**Hepatitis B/C serology, HIV test*****	7.5.9	X											
**Whole body plethysmography*****	7.5.10	X								X			
**Electrocardiogram (ECG) *****	7.5.10	X								X			
**Echocardiography*****	7.5.10	X								X			
**Testicular ultrasound*****	7.5.11	X											
**Abdominal ultrasound[7,***]**	7.5.12			X	X		X	X					
**Imaging (CT neck to pelvis))[8,***]**	7.5.13	X											

PERIODS	Name	SCREENING		TREATMENT – ARM A							EOT	FOLLOW-UP	
	Duration	14 days		16 weeks								2(-5) years	
VISITS	Title in eCRF	Screening		Visit 1	Visit 2	RA I	Visit 3	Visit 4	RA II	Visit 5	RA III	FU RA III – Yr 2	FU Yr 3-5[2]
	Time / Section	d-14 until d0	d0	d0-5 of cycle 1[1**]	d0-5 of cycle 2[1**]	d18-20 of cycle 2[1]	d0-5 of cycle 3[1**]	d0-5 of cycle 4[1**]	d18-20 of cycle 4[1]	d-6-0 of HCT[1,xx]	d25-35 after ASCT	*Every 3 mo*	*Every 6 mo*
**Imaging (gadolinium-enhanced brain MRI and response statement according to IPCG)**	7.5.14	X				X			X		X	X	X
**Central pathology[9]**	7.5.19	X											
**BM examination*** **	7.5.13	X											
**Slit lamp examination**	7.5.15	X				X[13]			X[13]		X[13]		
**CSF examination[10]**	7.5.16	X			(X)[15]	X[13]			X[13]		X[13]		
**MoCA and TMT-A/-B, QLQ[11]**	7.5.7/.8	X									X	X[12]	X[12]
**Neuropsychological battery[12]**	7.5.7	X									X	(X)	(X)
**HCT-CI**	7.5.20	X								X			
**Translational program[14]*****	7.6	X			X				X		X	(X)	(X)
**Concomitant medication**	6.3,6.4	X	X				X				X	X	X
**Adverse Events**	10	X	X				X				X	X	X
