# Supplementary material for: Optimizing MATRix as remission induction in PCNSL: de-escalated induction treatment in newly diagnosed primary CNS lymphoma
Source: BMC Cancer. 2022 Sep 10;22:971. doi: 10.1186/s12885-022-09723-w (PMC9464101; doi:10.1186/s12885-022-09723-w)
Supplement: Supplementary file 1 — Additional file 1: a. Flow Chart - control intervention (Arm A). b. Flow Chart - experimental intervention (Arm B) [file 12885_2022_9723_MOESM1_ESM.zip › FlowChart_ArmBR2.pdf]

Table 2 Visit schedule and assessments – Flowchart: Experimental Treatment – Arm B

**PERIODS**	**Name**	**SCREENING**		**TREATMENT – ARM B**						**EOT**	**FOLLOW-UP**	
		**14 days**		**12 weeks**							**2(-5) years**	
**VISITS**	**Title in eCRF**	**Screening**		**Visit 1**	**(clinical) RA I**	**Visit 2**	**Visit 3**	**RA II**	**Visit 4**	**RA III**	**FU RA III – Yr 2**	**FU Yr 3-5[2]**
	**Time / Section**	**d-14 until d0**	**d0**	**d0-1 of pre-phase[1]****	**d10-14 of pre-phase**	**d0-5 of cycle 1[1]****	**d0-5 of cycle 2[1]****	**d18-20 of cycle 2[1]**	**d-6-0 of HCT[1]**	**d25-35 after ASCT**	***Every 3 mo***	***Every 6 mo***
**Informed Consent[3]**	15.3	X										
**Inclusion / Exclusion Criteria**	4.2,4.3	X										
**Demographics, Medical History**	7.5.1,7.5.2	X										
**Pregnancy Test[4]**	7.5.3	X							X	X		
**Randomization**	5.2		X									
**Physical and neurological examination***	7.5.4	X		X	X[x]	X	X		X	X	X	X
**Vital signs***/ body height and weight**	7.5.5	X		X		X	X		X	X	X	X
**Performance status (Karnofsky and ECOG)**	7.5.6	X		X		X	X		X	X	X	X
**Laboratory tests (haematology[5], clinical chemistry[6])*****	7.5.9	X		X		X	X		X	X	X	X
**Creatinine, estimated GFR (MDRD) [6]**	7.5.9	X		X		X	X		X			
**LDH[6]**	7.5.9	X										
**Hepatitis B/C serology, HIV test*****	7.5.9	X										
**Whole body plethysmography*****	7.5.10	X							X			
**Electrocardiogram (ECG) *****	7.5.10	X							X			
**Echocardiography*****	7.5.10	X							X			
**Testicular ultrasound*****	7.5.11	X										
**Abdominal ultrasound[7,***]**	7.5.12			X		X	X					
**Imaging (CT neck to pelvis)[8,***]**	7.5.13	X										

PERIODS	Name	SCREENING		TREATMENT – ARM B						EOT	FOLLOW-UP	
		14 days		12 weeks							2(-5) years	
VISITS	Title in eCRF	Screening		Visit 1	(clinical) RA I	Visit 2	Visit 3	RA II	Visit 4	RA III	FU RA III – Yr 2	FU Yr 3-5[2]
	Time / Section	d-14 until d0	d0	d0-1 of pre-phase[1**]	d10-14 of pre-phase	d0-5 of cycle 1[1**]	d0-5 of cycle 2[1**]	d18-20 of cycle 2[1]	d-6-0 of HCT[1]	d25-35 after ASCT	*Every 3 mo*	*Every 6 mo*
**Imaging (gadolinium-enhanced brain MRI and response statement according to IPCG)**	7.5.14	X			(X)[x]			X		X	X	X
**Central pathology[9]**	7.5.19	X										
**BM examination*** **	7.5.13	X										
**Slit lamp examination**	7.5.15	X			X[13]			X[13]		X[13]		
**CSF examination[10]**	7.5.16	X			X[13]			X[13]		X[13]		
**MoCA and TMT-A/-B, QLQ[11]**	7.5.7/.8	X								X	X[12]	X[12]
**Neuropsychological battery[12]**	7.5.7	X								X	(X)	(X)
**HCT-CI**	7.5.20	X							X			
**Translational program[14]*** **	7.6	X			X			X		X	(X)	(X)
**Concomitant medication**	6.3,6.4	X	X	X						X	X	X
**Adverse Events**	10	X	X	X						X	X	X

RA= response assessment; d= day; mo= months; yr= year; EOT= End of study treatment; for additional details see corresponding numbering;

* Physical examination is recommended to be performed according to the flow chart; detailed findings concerning these examinations must only be documented in the eCRF at screening. At other visits, in case of clinically relevant abnormal findings, the investigator has to document an AE on the AE-page in the eCRF

** Interval of treatment administration

*** not to be documented in the eCRF

x Arm B: Thorough physical and neurological examination, documentation in eCRF mandatory; if any suspicion of progression, subsequent gadolinium-enhanced brain MRI must be performed prior to visit 2

xx d-6 of HCT matches d22 of last cycle of MATRix

1. Deviations ± 5 days are allowed, max. delay of therapy 4 weeks (additional gadolinium-enhanced brain MRI in case delay exceeds 2 weeks. Restart of treatment only in case of at least SD)
2. Also after termination of study, follow up every 6 months is recommended for yr3-yr5; annual follow up is recommended > yr5 for evaluation of overall survival and late toxicities
3. Informed consent must be obtained prior to any study specific (screening) examination
4. Serum beta-hCG
5. Haematology: white blood count (WBC), neutrophils, haemoglobin, and platelets
6. Blood chemistry: creatinine, total bilirubin, ALAT, ASAT, LDH (and gamma-GT only performed at screening); LDH, GFR (MDRD) and creatinine have to be documented in eCRF
7. Ultrasound of pericardium, pleura, abdomen to exclude third space fluid
8. If CT is suspicious at diagnosis: adequate further diagnostics by investigator’s decision, e.g. FDG-PET, biopsy
9. Results on central pathology will not be documented in the eCRF. At the end of the study, they will be converged with the clinical database
10. Only performed after excluding increased intracranial pressure by brain MRI; cytology, FACS and protein examination
11. EORTC QLQ-C30, -BN20; beginning with EOT every 12 months during follow-up period
12. Beginning with Follow-up MoCA and TMT-A and -B every 12 months as screening tests; subsequent neuropsychological battery only if 21-26 points in MoCA irrespective of TMT-B result, if ≥27 points in MoCA and PR (percentile) <10 in TMT-B (dependent on reference value regarding age and education, see Appendix 20.9.1) or if MoCA result deviates ≥4 points in comparison to previous test irrespective of TMT-B result. Take note: 2 different versions of neuropsychological battery exist ‘version 1’ and ‘version 2’). With beginning of screening and until end of study use the one assigned to the patient in the respective randomization process for neuropsychological testing (for details see 5.2)
13. Only performed if positive at diagnosis, examination until results are negative
14. Additional biological specimen (2 x EDTA tubes à 9 mL; 3 x streck tubes à 9 mL; 1 x serum tube à 9 mL; 1 x CSF Sarstedt tube à 4-5 mL,) has to be taken before IMP administration during screening period, at Visit 2 for Arm A/ at RAI for Arm B, at RAII, at EOT and only in case of relapse during follow-up period for translational research program. Only during screening period extra biological specimen (1 x bone marrow à 5-10 mL in EDTA) has to be obtained. Respective collecting and shipping material will be provided by the University Hospital Freiburg. For details on translational program and sample handling/ logistics see section 7.6 and respective study-specific “sample handling manual”.
15. Only performed if positive at screening and if patient receives the additional lumbar puncture at visit 2 as part of the translational program
